# Diagnosis, management and therapeutic strategies for congenital long QT syndrome

**DOI:** 10.1136/heartjnl-2020-318259

**Published:** 2021-05-26

**Authors:** Arthur A M Wilde, Ahmad S Amin, Pieter G Postema

**Affiliations:** Heart Centre, Department of Cardiology, Amsterdam Universitair Medische Centra, Amsterdam, The Netherlands

**Keywords:** tachycardia, ventricular, ventricular fibrillation, genetic services

## Abstract

Congenital long QT syndrome (LQTS) is characterised by heart rate corrected QT interval prolongation and life-threatening arrhythmias, leading to syncope and sudden death. Variations in genes encoding for cardiac ion channels, accessory ion channel subunits or proteins modulating the function of the ion channel have been identified as disease-causing mutations in up to 75% of all LQTS cases. Based on the underlying genetic defect, LQTS has been subdivided into different subtypes. Growing insights into the genetic background and pathophysiology of LQTS has led to the identification of genotype–phenotype relationships for the most common genetic subtypes, the recognition of genetic and non-genetic modifiers of phenotype, optimisation of risk stratification algorithms and the discovery of gene-specific therapies in LQTS. Nevertheless, despite these great advancements in the LQTS field, large gaps in knowledge still exist. For example, up to 25% of LQTS cases still remain genotype elusive, which hampers proper identification of family members at risk, and it is still largely unknown what determines the large variability in disease severity, where even within one family an identical mutation causes malignant arrhythmias in some carriers, while in other carriers, the disease is clinically silent. In this review, we summarise the current evidence available on the diagnosis, clinical management and therapeutic strategies in LQTS. We also discuss new scientific developments and areas of research, which are expected to increase our understanding of the complex genetic architecture in genotype-negative patients, lead to improved risk stratification in asymptomatic mutation carriers and more targeted (gene-specific and even mutation-specific) therapies.

## Introduction

Congenital Long QT Syndrome (LQTS), as the name implies, is characterised by a prolonged QT interval on the ECG, in the absence of structural heart disease and external factors such as a variety of drugs.^1–3^ LQTS was first described in the 1950s of the previous century, initially in a family with deafness (ie, Jervell and Lange-Nielsen syndrome). A few years later, in the early 1960s, patients with a similar ECG abnormality but without deafness were described (ie, Romano-Ward syndrome). Although initially this disease was subdivided into these two entities, later in time the more general terminology LQTS was used, and more recently (1995–1996), it became clear that, based on the underlying genetic defect, a further subdivision into distinct subtypes is pertinent. The various milestones in the over 60 years history of this disease are nicely summarised in a recent personal review by Dr Schwartz.^3^

### Diagnosis

**Figure 1:**
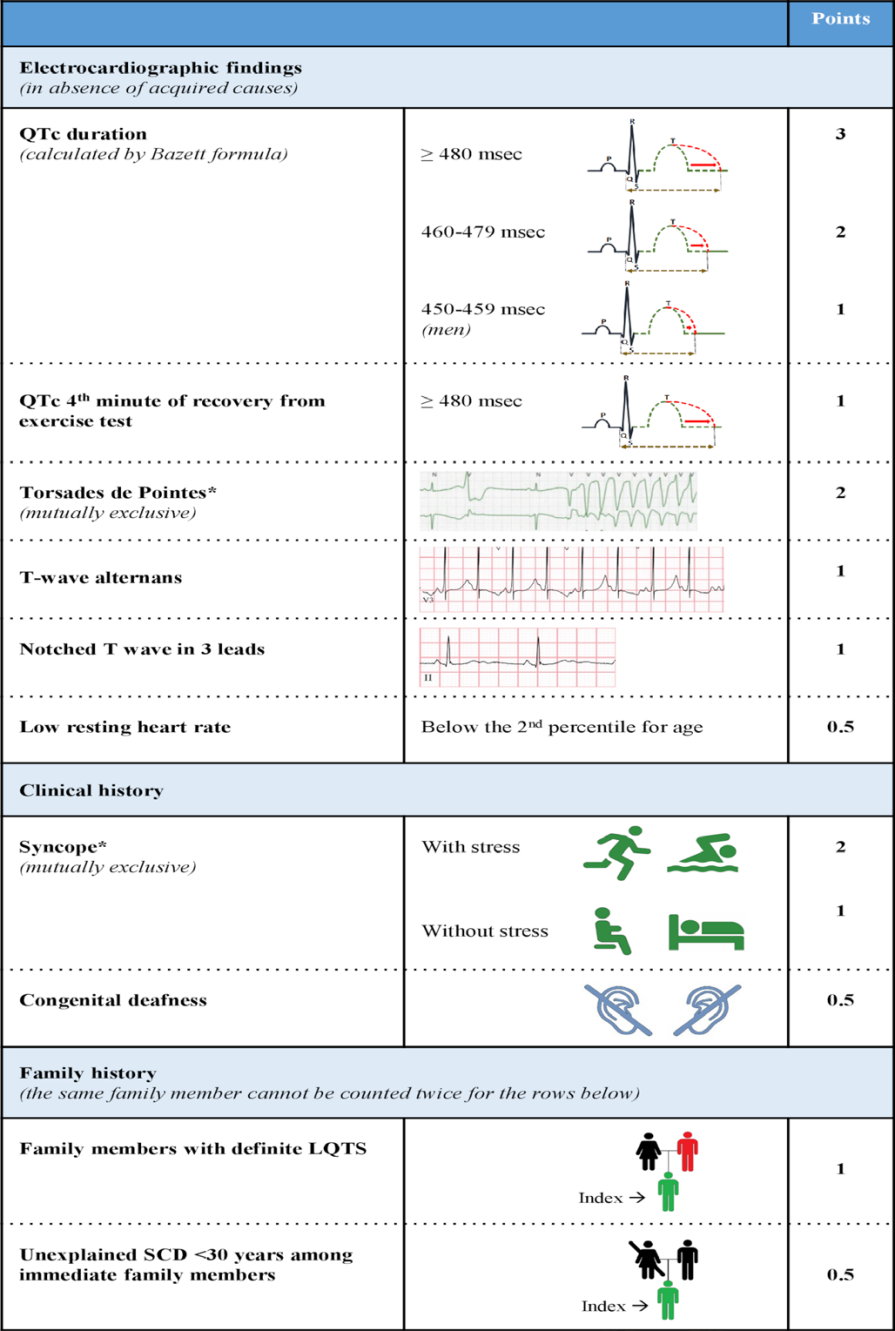
Diagnostic criteria for long QT syndrome (LQTS) (the ‘Schwartz-score’). Definite LQTS is defined by an LQTS score ≥3.5 points, intermediate probability of LQTS by an LQTS score of <3.5 and >1 and a low probability of LQTS by ≤1 point. In the family history rows, the same family member cannot be counted in both categories.

**Figure 2:**
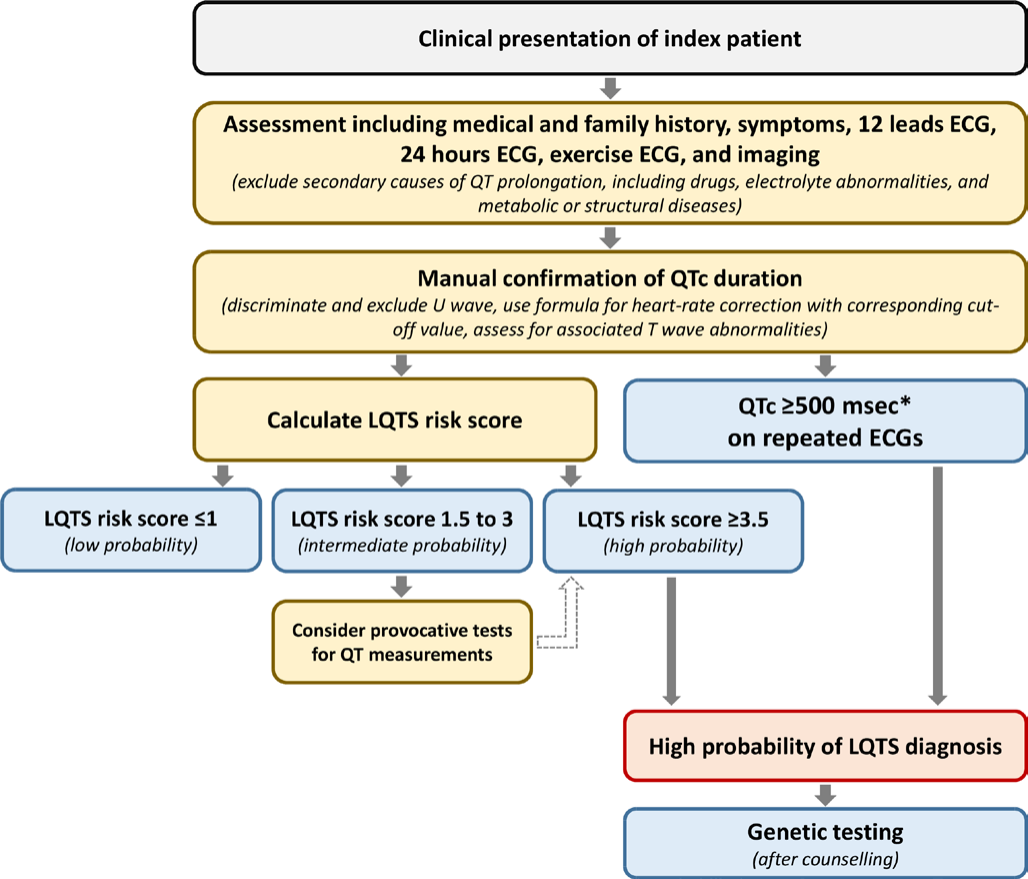
Schematic flow chart for the diagnosis LQTS. Flow chart for the diagnosis of LQTS following the Heart Rhythm Society/European Heart Rhythm Association/Asian Pacific Heart Rhythm Society consensus document from 2013.^3^ The LQTS risk score (ie, the ‘Schwartz score’) is presented in figure 1. *QTc calculated by Bazett formula (QTc=QT/√RR). LQTS, long QT syndrome; QTc, corrected QT interval.

The diagnosis of LQTS relies on the heart rate corrected QT interval (QTc) and on a number of other electrocardiographic parameters as well as elements obtained by history taking (eg, symptoms and family history). Together they form the LQTS probability or Schwartz score, where a score of ≥3.5 points indicates a high probability of LQTS (figures 1 and 2).^2 3^ Genetic information is not part of the Schwartz score but an individual with a pathogenic variant also fulfills the current diagnostic criteria for LQTS.^3^ It is important to realise that the QTc of individuals with pathogenic variants and normal healthy controls significantly overlap, indicating that a single QTc will never be able to distinguish all non-LQTS ECGs from all LQTS ECGs.^4 5^ It is also important to realise that there are different methods to measure the QT interval, which need different cut-off values, and that the formulas for heart rate correction are imperfect and also result in different cut-off values.^5^ In addition, although the U-wave can also be abnormal in LQTS patients, the U-wave should not be included in the QT assessment.^6^ Based on ECGs of a large number of genotyped LQTS patients and their family members not carrying the familial variant, we designed an online calculator with information on the likelihood that LQTS is present based on the calculated QTc (https://www.qtcalculator.org).^5^ In the past years, additional tools to more reliably assess LQTS on the ECG have been developed,^7–9^ including the use of artificial intelligence in establishing the diagnosis.^10^

### Genotyping and genotype–phenotype correlations

In the last 25 years, 17 genes have been associated with LQTS. However, a recent analysis, based on an approach using gene and disease specific metrics designed by the Clinical Genome Resource (ClinGen), reclassified a number of these genes to limited or disputed evidence.^11^ This approach left seven genes with definitive or strong evidence for causality (table 1).^11^ These remaining genes all encode for ion channels involved in cardiac repolarisation, their modulatory subunits or proteins regulating or modulating the function of ion channels. A positive genotype result, which is obtained in up to 75% of individuals with a clear phenotype, is of importance because it significantly contributes to risk and different aspects of the treatment strategy.^2 3^

**Table 1:** Classification of genetic evidence by the Clinical Genome Resource (ClinGen) for genes previously associated with LQTS

Gene	Protein	Level of evidence
*AKAP9*	A kinase anchor protein 9	Disputed
*ANK2*	Ankyrin-2	Disputed
*CACNA1C*	Calcium voltage-gated channel α1c subunit	Moderate
*CALM1*	Calmodulin-1	Definitive
*CALM2*	Calmodulin-2	Definitive
*CALM3*	Calmodulin-3	Definitive
*CAV3*	Caveolin-3	Limited
*KCNE1*	Potassium voltage-gated channel subfamily E regulatory subunit 1	Disputed
*KCNE2*	Potassium voltage-gated channel subfamily E regulatory subunit 1	Disputed
*KCNH2*	Potassium voltage-gated channel subfamily H member 2	Definitive
*KCNJ2*	Potassium voltage-gated channel subfamily J member 2	Limited
*KCNJ5*	Potassium voltage-gated channel subfamily J member 5	Disputed
*KCNQ1*	Potassium voltage-gated channel subfamily Q member 2	Definitive
*SCN4B*	Sodium voltage-gated channel β subunit 4	Disputed
*SCN5A*	Sodium channel voltage-gated α subunit 5	Definitive
*SNTA1*	Syntrophin α1	Disputed
*TRDN*	Triadin	Strong

*See for more details ref 11.

LQTs, long QT syndrome.

**Figure 3:**
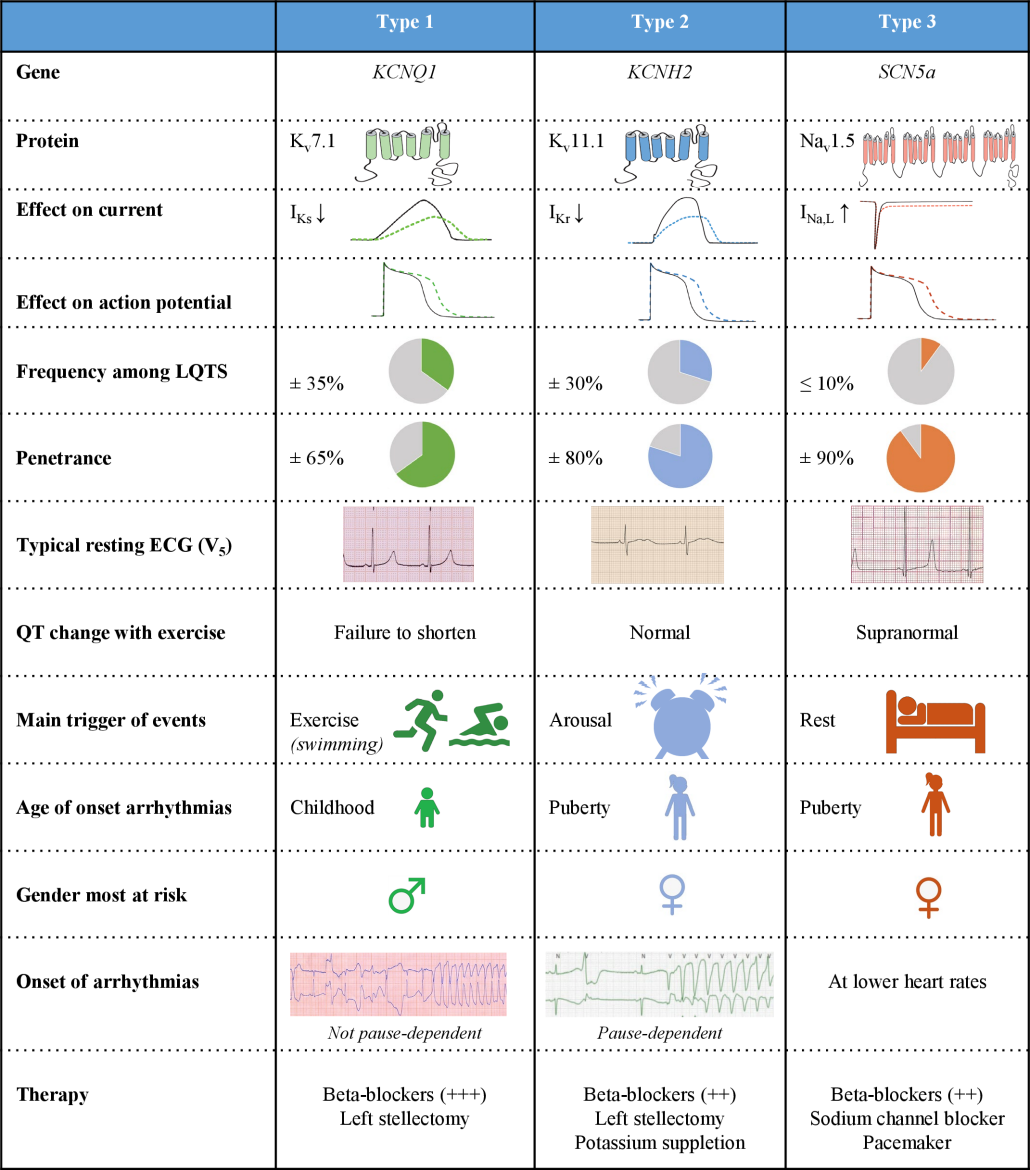
Genotype–phenotype relationship for the three most important subtypes, types 1, 2 and 3. See text for further explanation. *I*_Kr_, rapidly activating delayed rectifier potassium current; *I*_Kr_, rapidly activating delayed rectifier potassium current; *I*_Na, L_, late sodium current; LQTS, long QT syndrome; ↓, decrease; ↑, increase; +, therapeutic effect size of β-blocker therapy.

**Figure 4:**
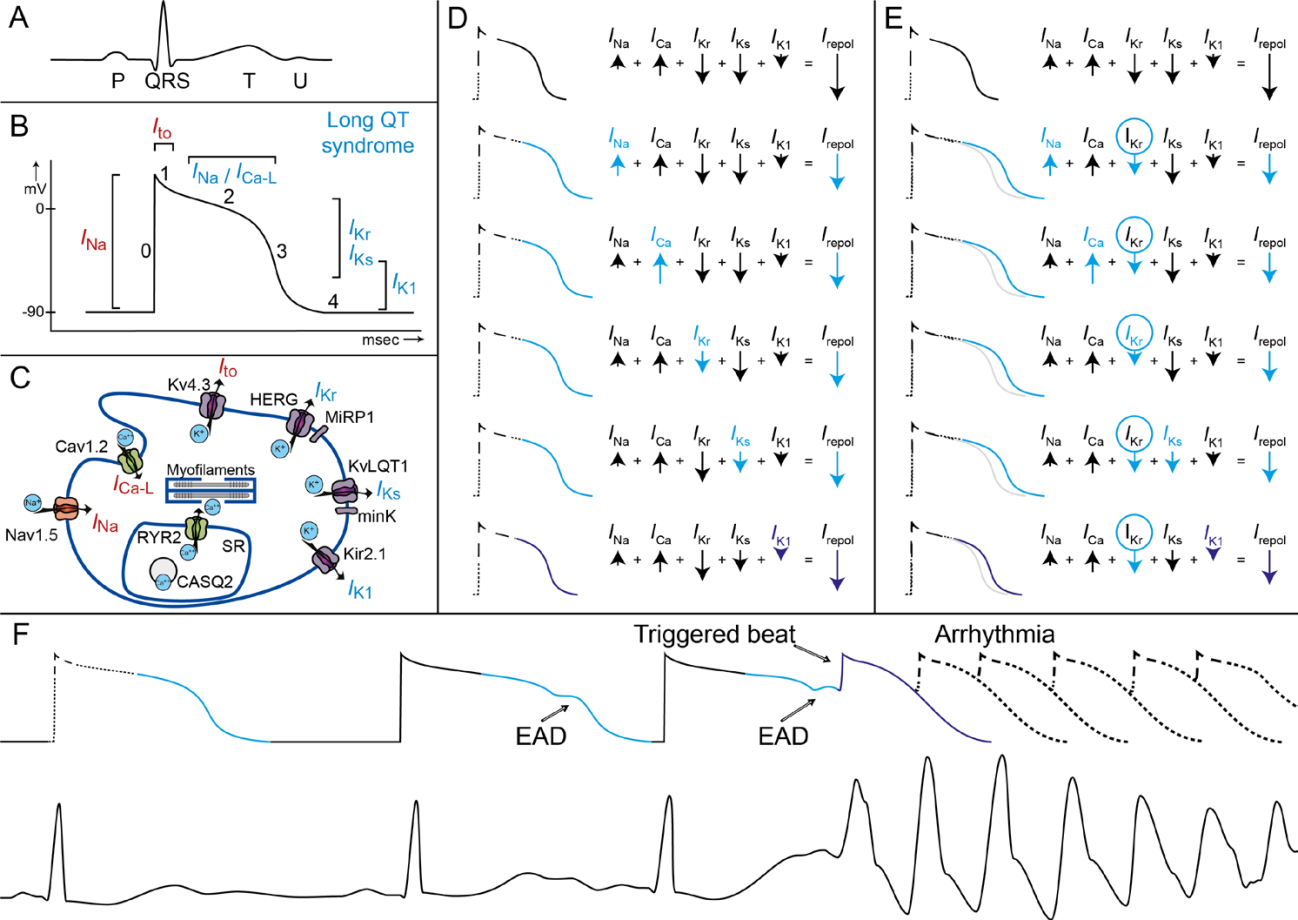
Pathophysiological mechanisms underlying LQTS and associated arrhythmia. Figure parts A–C show the illustrations of (A) an ECG with a normal P-QRS-TU complex, (B) a corresponding ventricular action potential with the different cardiac ion currents (I) and (C) a cardiomyocyte with different ion channels, subunits and their currents. In long QT syndrome, the phase 2 late sodium inward current (*I*_Na_) the slow calcium inward current (*I*_Ca-L_), or the potassium rectifier currents during phase 3 and phase 4 (*I*_Kr_, *I*_Ks_, *I*_K1_) are involved. (D and E) In these illustrations, the action potential prolongation caused by a decrease in net repolarising current (*I*_repol_) by changes in one of the repolarising currents in blue is shown. (D) If any of the currents in repolarisation is altered (eg, by a LQT3 *SCN5A* mutation with more net *I*_Na_ or a LQT1 *KCNQ1* mutation with less net *I*_Ks_), the ventricular action potential (and the corresponding QT interval) will lengthen. (E) When a second hit on net repolarising current is introduced, for example, by (further) decrease of *I*_Kr_ current due to the use of certain drugs, the ventricular action potentials and the QT interval will further lengthen. It can be appreciated that a loss of function of *I*_K1_ only has a minor effect on the action potential duration (purple). (F) In this illustration, the prolonged ventricular action potential durations correspond with prolonged QT intervals, which are further challenged by changes in heart rate and proceed to early after depolarisation (EAD). These EADs finally result in a triggered beat and the onset of malignant ventricular arrhythmia (Torsades de Pointes). LQTS, long QT syndrome.

Indeed, specific genotype–phenotype relationships have been described for the three most common subtypes: LQTS types 1, 2 and 3 (figure 3). The first two subtypes (LQT1 and 2) are based on functional loss-of-function variants in the potassium channel genes *KCNQ1* and *KCNH2*, respectively. These genes respectively encode for the slow and rapid delayed rectifier current *I*_Ks_ and *I*_Kr_, and a smaller amplitude of this current leads to prolongation of the QT interval (figure 4A–D). LQTS type 3 is based on gain-of-function variants in *SCN5A*, the gene encoding the fast inward cardiac sodium current (*I*_Na_). Gain of function relates to an increased amplitude of the late sodium inward current (during the plateau phase), which will also lead to prolongation of the action potential (figure 4D).

The age of onset of arrhythmias is typically younger in LQT1 patients and in particular LQT1 males are at risk, whereas most LQT2 and LQT3 patients who become symptomatic experience their first symptoms around puberty and here particular females are at risk (figure 3). Also, the morphology of the ST-T segments is rather specific for the three subtypes, and the genotype can be accurately predicted with these characteristic features.^12 13^ Furthermore, each genotype also has specific triggers for arrhythmic events and rather specific electrocardiographic features of the onset of the arrhythmias (figure 3).^14–16^ The arrhythmias in LQTS (figure 4F) originate from the last part of the ventricular action potential where severe action potential prolongation results in early afterdepolarisations that at one instant reach threshold for subsequent fast sodium inward current and a trigger beat that then degenerates into fast polymorphic ventricular arrhythmia: Torsades de Pointes and ventricular fibrillation. Adrenergic triggers, particularly exercise and swimming, are the most important trigger for arrhythmic events in LQT1,^15^ whereas in LQT2 sudden arousal (ie, auditory stimuli) is predominant (figure 3).^14 15^ Events in LQT3 occur most frequently in rest.^15^ These gene-specific characteristics not surprisingly associate with a high response to β-blockers of LQT1 patients, although also in LQT2 and LQT3 patients β-blockers constitute the first line of therapy. LQT3 patients are most sensitive to (late) sodium channel current blockers.^17 18^ Because of the genotype-specific features (figure 3) with impact on prognosis and therapy, genetic testing has become an integral part of the diagnosis and management of LQTS patients.^2 3^

### Clinical management and treatment strategies

**Figure 5:**
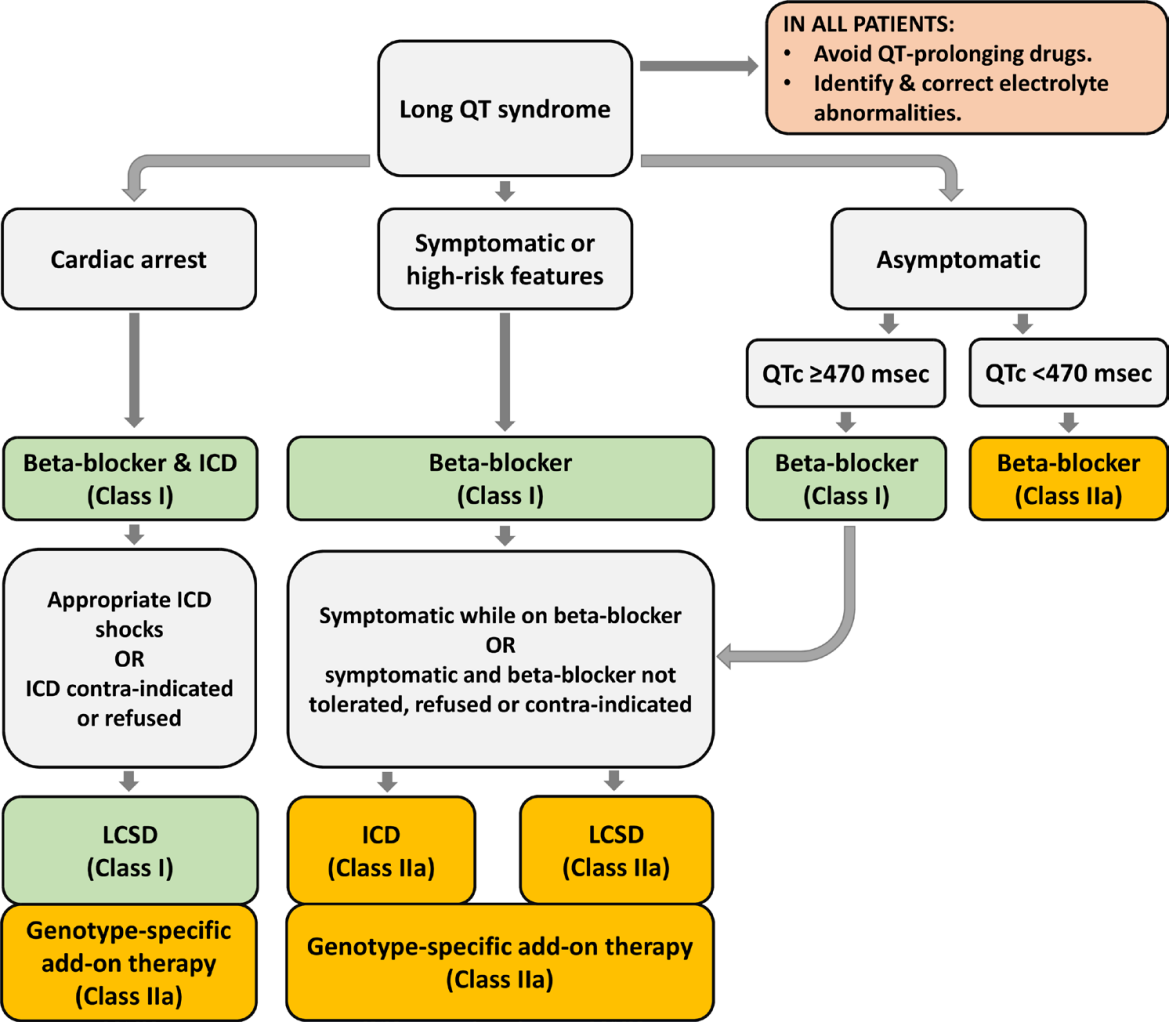
Schematic flow chart for the therapeutic choices in LQTS. Flow chart for therapeutic choices in LQTS following the HRS/EHRA/APHRS consensus document from 2013.^3^ See text for further discussion. ICD, internal cardioverter defibrillator; LQTS, long QT syndrome.

As indicated, the cornerstone of management of LQTS patients is ß-blocker therapy (figure 5). The non-selective ß-blockers nadolol and propranolol have been advocated as the most effective drugs.^19 20^ The efficacy of propranolol was questioned,^20^ but we believe that the published data from the LQTS registry^18^ are ‘contaminated’ with the inclusion of symptomatic newborns, which is a severe condition with failure of almost every therapeutic attempt. This includes propranolol, the only available β-blocker in liquid form and therefore exclusively given in this patient group.^21^ Metoprolol and atenolol are less effective and should be avoided, at least in symptomatic patients.^19^ The antiarrhythmic effect of β-blockers is due to the prevention of early after depolarisations by blocking the adrenergic driven boost of calcium current. Only propranolol reduces the QTc to some extent by blocking late sodium inward current.^22^ There is no doubt that all symptomatic patients should be treated. However, although we have shown that active treatment is installed in the vast majority of presymptomatically screened individuals,^23^ the need for treatment in asymptomatic individuals is less determined (see further). This is not a trivial issue because the latter group expands rapidly with active cardiogenetic programmes.

Additional pharmacological therapy consists of blockers of the late sodium inward current (ie, mexiletine, flecainide and ranolazine). As mentioned previously, LQT3 patients in particular are responsive to sodium channel blockers,^17 18^ but in a recent study, mexiletine has also been shown to reduce the QTc in LQT2 patients.^24^ Sodium channel blockers, which also block the fast sodium inward current, may, while shortening QTc, provoke a Brugada ECG pattern however, which thus warrants evaluation.^25^ The latter is particularly relevant in patients with SCN5A variants with both gain-of-function and loss-of-function characteristics.^26^ Potassium suppletion has been shown to be particularly effective in LQT2 because the conductance slope of *I*_Kr_, the affected current in LQT2, heavily depends on the extracellular potassium level (the higher, the steeper).^27 28^ Still, also the *I*_Ks_ and *I*_K1_ current are critically dependent on extracellular potassium levels^29 30^ and may thus well contribute to increased QTc intervals and an increased risk for arrhythmia in states of low plasma potassium levels.

New pharmacological therapy is emerging, but the target population becomes more and more specific. In patients with KCNH2 trafficking defects (ie, mutated protein products that do not travel adequately to the cell membrane), lumacaftor has been shown to shorten the QTc significantly.^31^ Lumacaftor is a drug that has been shown to impact on intracellular trafficking of mutated protein products.

**Figure 6:**
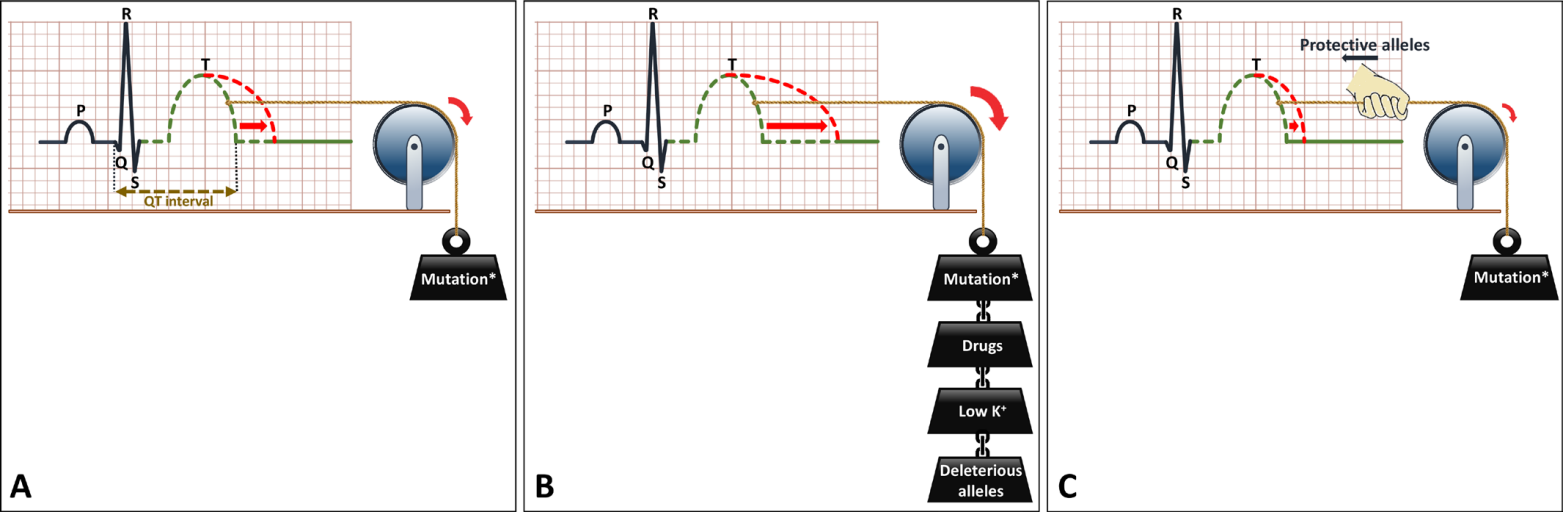
Schematic representation of the effects of genetic and environmental factors in LQTS. (A) LQTS-associated mutation causes prolongation of the QT interval on the ECG. (B) Environmental factors such as certain drugs (which decrease repolarisation reserve) or hypokalaemia, or genetic factors (ie, deleterious alleles) act in a conjoint manner with the LQTS-associated mutation to further prolong the QT interval. (C) Protective alleles counteract the effects of the mutation and reduce the extent of QT prolongation. The presence of deleterious and/or protective alleles may underlie, at least partially, the variable expressivity in LQTS. LQTS, long QT syndrome.

Another important pharmacological issue for the prevention of malignant arrhythmia in all LQTS patients is the avoidance of certain drugs that decrease repolarisation reserve (figure 4E). Also, electrolyte disorders, most importantly hypokalaemia, should be avoided. QTc prolongation may be more severe through the conjoint effect of drugs and/or hypokalaemia and a LQTS-causing mutation (figure 6A, B). The Arizona Center for Education and Research on Therapeutics, currently known as CredibleMeds, has created a website (www.QTdrugs.org) for this purpose. This website is widely used across the world but has limitations that should be acknowledged.^32^

The next level of therapy is left cardiac sympathetic denervation (LCSD). β-blocker breakthrough events are not rare, and additional therapy is thus frequently required. LCSD is the first choice in β-blocker resistant patients (figure 5).^3 33 34^ Indeed, the HRS/EHRA/APHRS expert consensus has recommended for LCSD to be performed in high-risk patients with a diagnosis of LQTS in whom: (1) ICD therapy is contraindicated or refused and/or (2) β-blockers are either not effective in preventing syncope/arrhythmias, not tolerated, not accepted or contraindicated (class I), and it may be useful in patients with a diagnosis of LQTS who experience breakthrough events while on therapy with β-blockers/ICD (class IIa). LCSD is particular effective in LQT1 patients, but also LQT2, LQT3, gene-elusive LQTS and patients with multiple mutations (eg, compound heterozygous) may respond well on LCSD.^3 35^

Device therapy is the final level of therapy in LQT patients with ß-blocker and/or LCSD breakthrough cardiac events or in patients with an anticipated high risk for arrhythmic events. In patients who survived a cardiac arrest, ICDs have a class 1 recommendation (on top of ß-blockers; figure 5), with a possible exception of LQT1 patients who were not yet diagnosed (and, as a consequence, not treated with ß-blockers) at the time of their arrest. ICDs are well accepted and have shown clear benefit in primary and secondary sudden cardiac death prevention. However, ICD therapy does not come without disadvantages. In a systematic review with an emphasis on ‘ICD-harm’ in patients with inherited arrhythmia syndromes, the yearly rate of complications in LQT patients was 7.0% (95% CI 4.4 to 9.7) on top of a yearly 3% of inappropriate shocks (2.0–3.6).^36^ Appropriate therapy was 1.9% and 6.8% in, respectively, primary and secondary prevention patients.^36^

Pacemaker therapy has not extensively been tested in LQTS but may be beneficial in particular in LQT2 patients where arrhythmias are almost exclusively pause dependent.^16^ Likewise, pause-dependent algorithms (‘rate smoothing’) have successfully been applied in single cases.^37^ In LQT3, QTc prolongation is particularly evident at lower rates, so also in this subentity, background pacing at a higher than spontaneous rate, which is actually low in LQTS patients, may be beneficial. The latter has been well shown in a large family with a specific LQT3 variant.^26^ Furthermore, in the rare but severe Jervell Lange-Nielsen type LQTS, atrial pacing has been shown to be very effective already at young age.^38^ Pacing at a higher rate (>100/min) has also been shown to be temporarily effective, by preventing pauses, in the acute phase of an arrhythmic storm.^39^

### Knowledge gaps or future research opportunities

The advancement of knowledge in the LQTS field has been tremendous with the change from the description of individuals with a electrocardiographic curiosity, many of whom died suddenly, to a genotype-specific approach of the different disease subtypes.^1^ This development has led to more sophisticated risk stratification, genotype-specific treatment modalities and significantly reduced mortality.^1 2 18 28 40^ However, further optimisation of treatment strategies is pertinent, in particular in two specific patient categories. The first is a small group of treatment resistant severe LQTS, most often consisting of neonates with specific SCN5A mutations (eg, R1623Q),^41^ CALM mutations,^42^ or Triadin mutations,^43^ or patients with double mutations. These patients not rarely die despite aggressive treatment on all levels, including ICDs. New, more effective drugs are needed for this small subgroup. The second group is much larger and still expanding because of the proactive cardiogenetic counselling programmes around the world. Many of these presymptomatic counselled individuals have a normal or only marginally prolonged QTc and one may question whether they do need treatment and strict preventive measures at all. More sophisticated risk stratification in this group of patients is desperately needed.

Furthermore, and relevant for all patients, it is important to note that adherence to ß-blocker therapy is overall poor. In a study from New Zealand, adequate adherence was reported in only 50% of LQT patients.^44^ Side effects are frequent and contribute, with illness perception and believe in medication, to non-adherence.^44 45^ Obviously, this has potentially serious impact on the freedom from arrhythmic events. Non-compliance has been shown, together with the unintentional use of QT-prolonging drugs, to be the major component in ‘ß-blocker failures’ in LQT1 patients.^46^ LCSD as a stand-alone therapy may also be an option as shown by an observation in 64 patients most of whom suffered from severe ß-blocker side effects.^35^ In almost 3 years of follow-up, three patients from this cohort with a higher baseline risk for events had an arrhythmic (but non-lethal) event after LCSD.

Finally, despite all progress in genetic screening methods, the yield of finding a potential pathogenic variant in a LQTS patient with a clear phenotype is at most 75%–80%.^2^ However, in dedicated cardiogenetic clinics the yield was lower, around 50%, with these higher values only in earlier years and lower values in more recent years because of a more lenient case selection in recent years.^47^ Important additional scientific inquiries in this field are the unravelling of the genotype in the 25%–30% genotype elusive cases and the role of additional genetic and other factors in determining the arrhythmia risk. For example, comorbidities like hypertension may aggravate the LQTS phenotype by deleterious effects of the interaction between hypertrophy and the mutation.^48^ Furthermore, recent data show evidence for a more complex polygenic architecture in genotype-negative patients,^49^ and preliminary evidence in single families has revealed evidence for the existence of both protective as well as deleterious alleles, which may modify the phenotype by, respectively, aggravating (figure 6B) or alleviating (figure 6C) the QT-prolonging effects of a LQTS-causing mutation.^50^ New genes, yet to be identified, may also form a partial explanation for the genetic underpinning in gene-elusive patients.

In summary, congenital LQTS is an inheritable entity characterised by a prolonged heart-rate corrected QT interval, and it associates with malignant arrhythmias at young age. It is caused by a decrease in repolarising cardiac ion currents in a complex polygenic composition and interacting with multiple other factors such as sex, age, comorbidities and triggers such as drugs. Therapy relies importantly on ß-blockers and lifestyle measures. More patient specific pharmacological and invasive therapies are available and under continuous development. A better understanding of its complex architecture paves the path to improving care for these patients and their families.
